# The effects of urolithin A on poly I:C-induced microglial activation

**DOI:** 10.3389/fncel.2024.1343562

**Published:** 2024-03-20

**Authors:** Yakum Benard Mingo, Lea Gabele, Niklas Lonnemann, Bert Brône, Martin Korte, Shirin Hosseini

**Affiliations:** ^1^Department of Cellular Neurobiology, Zoological Institute, Technische Universität Braunschweig, Braunschweig, Germany; ^2^Laboratory for Neurophysiology, Biomedical Research Institute, Hasselt University, Hasselt, Belgium; ^3^Helmholtz Centre for Infection Research, Research Group Neuroinflammation and Neurodegeneration, Braunschweig, Germany

**Keywords:** poly I:C, microglial activation, neuroinflammation, oxidative stress, urolithin A

## Abstract

Neuroinflammation can be triggered by various stimuli, including viral infections. Viruses can directly invade the brain and infect neuronal cells or indirectly trigger a “cytokine storm” in the periphery that eventually leads to microglial activation in the brain. While this initial activation of microglial cells is important for viral clearance, chronic activation leads to excessive inflammation and oxidative stress, which can be neurotoxic. Remarkebly, recent studies have shown that certain viruses such as influenza A virus, coronavirus, herpes virus and Epstein–Barr virus may be involved in the development of neurodegenerative diseases such as Parkinson’s disease, Alzheimer’s disease, and multiple sclerosis. Therefore, it is important to find therapeutic strategies against chronic neuroinflammation triggered by viral infections. Here, we investigated the effects of urolithin A (UA) on microglial activation *in vitro* induced by a viral mimetic, poly I:C, in a triple co-culture system of neurons, astrocytes and microglial cells. Immunocytochemistry was used to perform a comprehensive single-cell analysis of the morphological changes of microglia as an indicator of their reactive state. Treatment with UA significantly prevented the poly I:C-induced reactive state of microglia, which was characterized by increased expression of the microglial activation markers CD68 and IBA-1. UA restored the poly I:C-induced morphology by restoring microglial ramification. In addition, UA was able to reduce the release of the pro-inflammatory mediators CCL2, TNF-α, and IL-1β and showed a trend toward attenuation of cellular ROS production in poly I:C-treated cultures. Overall, this study suggests that UA as a component of a healthy diet may help prevent virus-induced neuroinflammation and may have therapeutic potential for future studies to prevent or treat neurodegenerative diseases by targeting the associated neuroinflammatory processes.

## 1 Introduction

Infectious diseases caused by pathogens such as viruses, bacteria, and parasites represent an increasing concern for the central nervous system (CNS) (Sahu and Ter, 2018). In particular, viral infections also pose potential threats to the CNS. Evidence is accumulating that both neurotropic and non-neurotropic viruses can cause acute and long-term neurological sequelae (Jang et al., 2009, 2012; Sadasivan et al., 2015; Hosseini et al., 2018, 2021; Li et al., 2019). These consequences include impairments in the structure and functions of neurons, reactivity of glial cells, neurodegeneration, as well as cognitive decline, and behavioral changes. While the underlying causes of most neurodegenerative diseases remain elusive, recent studies suggest that neurological defects triggered by viral infections may contribute to the development or exacerbation of neurodegenerative diseases.

For instance, *Bjornevik* et al. recently found that Epstein-Bar virus (EBV) infection in humans increases the risk of multiple sclerosis (MS) by 32-fold, making EBV one of the most important triggers for the development of MS (Bjornevik et al., 2022). In addition, *Hosseini* et al. have previously shown that the non-neurotropic H3N2 influenza A virus exacerbates Alzheimer’s disease (AD) symptomes in the APP/PS1 mouse model (Hosseini et al., 2021). These studies highlight the important role that viruses play in the pathophysiology of neurodegenerative diseases. Moreover, these diseases affect more than 50 million people worldwide, and there are currently no effective cures (Eshraghi et al., 2021). Therefore, there is a need for novel therapeutic approaches that target the underlying pathological mechanisms triggering the development of neurodegenerative diseases.

Microglia, the resident innate immune cells of the brain, make up about 10% of all cells in the brain (Prinz et al., 2021; Xu et al., 2021). They perform numerous functions in neurodevelopment, synaptic pruning, CNS homeostasis, immune sensing, and protection against invading pathogens. Because of their vital role in the CNS, microglial dysfunction has been shown to be an important factor in neurodegeneration (Perry and Holmes, 2014). In the case of neurotropic viral infections, microglia recognize invading viral pathogen-associated molecular patterns (PAMPs) via PAMP recognition receptors (PRRs), such as Toll-like receptor 3 (TLR3) for viral double-stranded ribonucleic acid (dsRNA) (Mogensen and Paludan, 2005; Town et al., 2006; Bowie and Unterholzner, 2008; Li et al., 2019). In response, ramified microglia, which constantly monitor the microenvironment with their highly motile processes and also perform cell debris removal by phagocytosis (Neumann et al., 2009), retract their processes, proliferate, and enlarge into activated and amoeboid phenotypes to mediate innate and adaptive immune responses (Streit et al., 1999; Sahu and Ter, 2018; Li et al., 2019; Edler et al., 2021). In addition, microglial cells in their reactive state can exhibit an intermediate morphology with partially retracted processes and produce pro- or anti-inflammatory cytokines depending on the microenvironment, whereas the amoeboid state with fully retracted processes mainly engages in phagocytosis but does not fulfill the same antigen-presenting and inflammatory functions as reactive microglia (Paolicelli et al., 2022; Vidal-Itriago et al., 2022). Moreover, microglial cells can also adopt different reactivity states in non-neurotropic viral infections. Indeed, excessive systemic inflammation induced by non-neurotropic viruses can disrupt the blood–brain barrier and trigger microglial priming via a “cytokine storm” without direct virus entry into the CNS (Sadasivan et al., 2015; Barbosa-Silva et al., 2021; Lima et al., 2022). Reactive microglial cells can serve opposing functions during viral infections. Initial activation is essential for mediating inflammatory and cytotoxic processes required for clearance of viral particles and virus-infected cells (Jang et al., 2012; Sadasivan et al., 2015). However, prolonged microglial reactivity, such as in chronic infections, can lead to neurotoxicity. This neurotoxic effect is mediated by the excessive release of pro-inflammatory cytokines and chemokines, such as tumor necrosis factor-alpha (TNF-α), interleukin-1 beta (IL-1β), IL-6, and microglia/monocyte chemoattractant protein (CCL2) (Li et al., 2019). CCL2, among other attractant proteins, recruits monocytes and other immune cells to support viral clearance and recovery, but this may exacerbate existing inflammation (Khalil et al., 2021).

Furthermore, viral infections have also been shown to trigger mitochondrial dysfunction in affected cells due to impaired mitophagy (Thangaraj et al., 2018; Foo et al., 2022; Pliss et al., 2022). Mitophagy is a selective form of autophagy in which damaged mitochondria are degraded and recycled for mitochondrial biogenesis. However, when disrupted, damaged mitochondria accumulate in cells, causing oxidative stress through increased release of reactive oxygen species (ROS) (Thangaraj et al., 2018; Eshraghi et al., 2021). Long-term exposure of neurons to pro-inflammatory cytokines and excessive ROS leads to neuronal damage that can trigger or exacerbate neurodegeneration (Kaul et al., 2001; Streit et al., 2004; Sahu and Ter, 2018; Li et al., 2019). Therefore, modulation of these viral mechanisms in microglia may be a useful strategy to prevent virus-triggered, long-lasting and therefore maladptive neuroinflammation and subsequent neuronal damage.

In the current study, urolithin A (UA) was used as a potential neuroprotective agent to prevent microglial activation associated with viral infections. Viral infection was mimicked by administration of polyinosine: polycytidylic acid (poly I:C), a synthetic TLR3-agonist that mimics dsRNA viral nucleic acids. Urolithins (A-D) are metabolites of intestinal bacteria from foods rich in ellagic acids and ellagitannins, such as pomegranate, berries, and nuts (D'Amico et al., 2021). Recent studies have reported the beneficial properties of UA on aging and various diseases by attenuating inflammation and improving mitochondrial function. Notably, UA prolongs the lifespan of *Caenorhabditis elegans* and has recently been shown to improve cognitive impairment and prevent neuronal apoptosis in the APP/PS1 mouse model of AD (Ryu et al., 2016; Gong et al., 2019). In addition, UA was recently shown to limit lipopolysaccharide (LPS)-induced microglial activation and reduce motor deficits in the MPTP (1-methyl-4-phenyl-1,2,3,6-tetrahydropyridine) mouse model of Parkinson’s disease (Qiu et al., 2022). However, the potential beneficial role of UA in the pathology of viral infections is still unknown. Here, we hypothesized that UA rescues poly I:C-induced microglial activation and attenuates excessive inflammation and oxidative stress in an *in vitro* model consisting of a co-culture of neurons, astrocytes, and microglia.

To investigate this hypothesis, a comprehensive single-cell analysis of microglial cells was performed in this study using structured illumination microscopy (SIM) with ApoTome. Our results showed that UA was able to prevent poly I:C-induced microglial activation and proliferation and limits the characteristic morphological shift of microglia from ramified to an intermediate reactive phenotype. In addition, UA suppressed the production of pro-inflammatory molecules (CCL2, TNF-α, and IL-1β) after poly I:C stimulation and showed a tendency to induce mitochondrial biogenesis by attenuating cellular ROS production in poly I:C-stimulated cultures. Overall, this study provides further insight into the potential benefits of urolithin A against neuroinflammatory processes induced by viral infections and may be useful for future studies on therapeutic strategies against various neurological complications associated with viral infections.

## 2 Materials and methods

### 2.1 Preparation of primary embryonic hippocampal culture

Primary embryonic hippocampal cultures were prepared as previously described (Michaelsen et al., 2010). Briefly, C57BL/6 J mice were decapitated at embryonic day 17.5 (E17.5), and hippocampi were carefully separated from cortices and dissociated in 1X trypsin/EDTA solution (Sigma, T3924) for 25 min at 37°C. Then, 7 × 10^4^ cells per well were plated in a 24-well plate on a poly-L-lysine coated 13 mm diameter coverslip and cultured for 21 days in Neurobasal medium (Invitrogen, 21,103,049) supplemented with N2 (self-made), B27 (Invitrogen, 17,504–001), and 0.5 mM L-glutamate (Invitrogen, 25,030–024), to obtain a dissociated culture of primary neurons and astrocytes.

### 2.2 Preparation of triple co-culture of neurons, astrocytes and microglia

The triple co-culture model was prepared to contain microglia, neurons and astrocytes in order to obtain microglial cells in a more physiological state than in microglia monocultures, as microglial cells in primary pure microglia cultures do not exhibit a typical ramified morphology (Culbert et al., 2006). Briefly, primary embryonic hippocampal cultures containing neurons and astrocytes were first prepared as described above. Pure microglia were then isolated at 14 days *in vitro* (DIV) and seeded onto the neuron-astrocyte cultures at DIV 21 (Hernangómez et al., 2012). For this purpose, cerebral cortices from C57BL/6 J mice were harvested after the third to fifth postnatal day in cold 1X HBSS medium (Gibco, 14,185–045) and centrifuged at 2000 rcf for 5 min at 4°C. Cells were cultured for 14 days in DMEM medium (Gibco, 6,195–026) containing 10% fetal calf serum (FCS) (Capricorn-scientific, FCS-62A) and 1% penicillin/streptomycin (Gibco, 15,070–063), preserving only glial cells. Microglia were then isolated by shaking the flasks in a shaker incubator at 200 rpm at 37°C for 3 h. 3 × 10^4^ microglia were plated onto the embryonic hippocampal culture per well and allowed to adhere overnight. Triple co-cultures were treated 48 h after microglia plating. For pure microglia monocultures, microglial cells were plated in 96-well plates at a density of 6 × 10^4^ cells/well as previously described (Lonnemann et al., 2020). The purity of the cultures was determined by immunostaining for IBA-1, NeuN and GFAP. And all cultures contained these three cell populations.

### 2.3 Treatment of triple co-cultures

**Figure 1:**
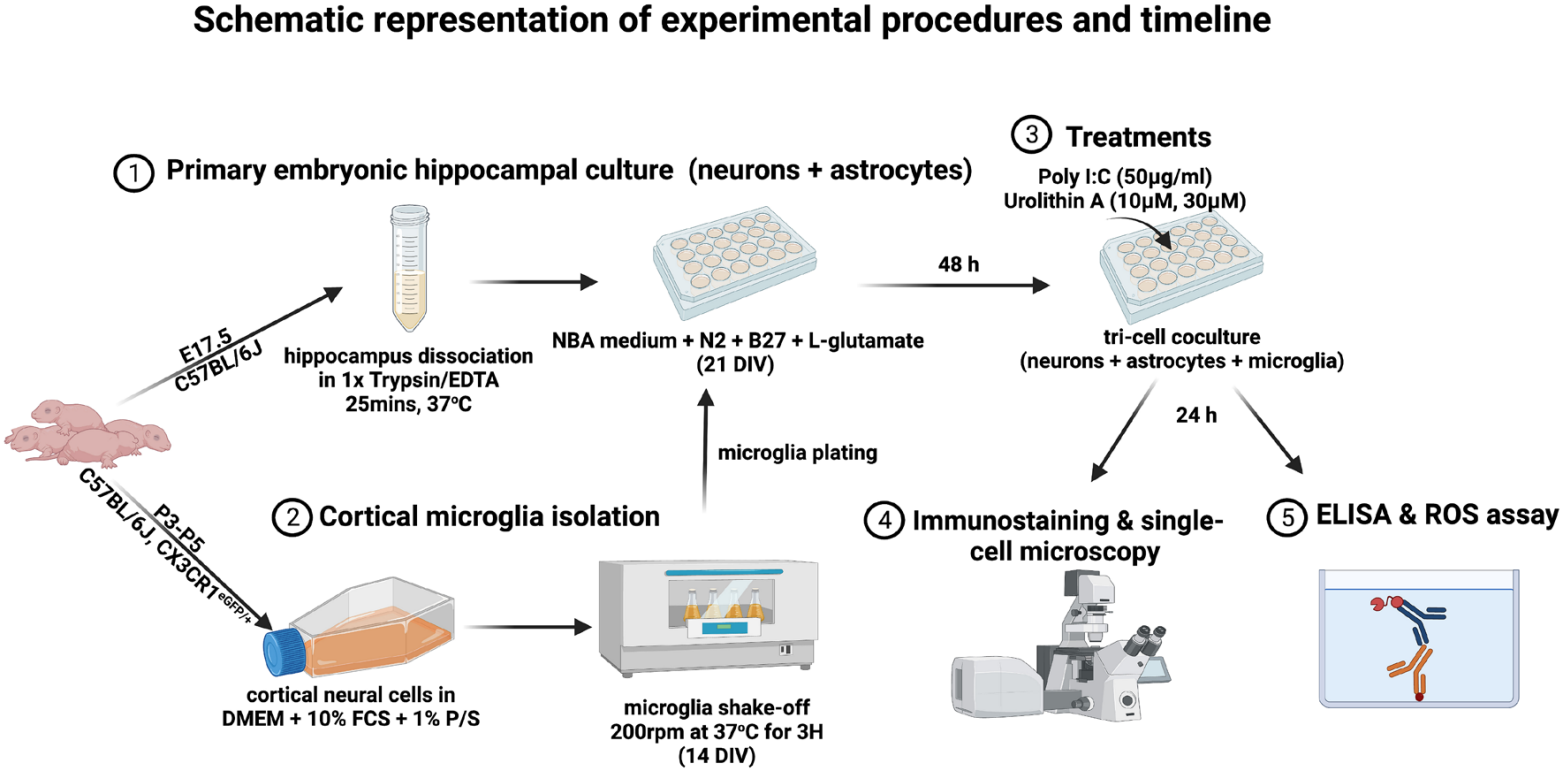
The timeline of the experimental procedures is displayed (created with BioRender.com).

A synthetic TLR3 agonist, polyinosinic: polycytidylic acid (poly I:C), was used to mimic TLR3 stimulation of cells during dsRNA viral infections. DIV 14 microglia were added to primary embryonic hippocampal cultures (DIV 21) and 48 h later treated with 50 μg/mL poly I:C (Merck KGaA, 42,424–50-0) alone or with the different concentrations of UA (10 μM and 30 μM) (MedChem Express, HY-100599). All reagents were prepared in Neurobasal medium (Invitrogen, 21,103,049), supplemented with N2 (self-made), B27 (Invitrogen, 17,504–001), and 0.5 mM L-glutamate (Invitrogen, 25,030–024). Culture medium without poly I:C or UA was added to the control groups. At 24 h after treatment, cell supernatants were collected for cytokine analysis and cells were processed for immunocytochemistry. For cytokines analysis, in another series of experiments, pure microglia cultures were treated with poly I:C and UA according to exactly the same protocol as described above (Figure 1).

### 2.4 Immunocytochemistry

Cells were fixed in cold 4% paraformaldehyde (PFA) for 15 min at room temperature and washed 3 times with 1X PBS for 5 min on a shaker. Cells were then permeabilized with 0.2% Triton X-100 (AppliChem, A4975,0100) for 30 min on a shaker and blocked in 1% bovine serum albumin (BSA) for 5 min to prevent non-specific binding. Cells were then stained with primary antibodies and incubated for 1 h at 30°C in a humidity box. The primary antibodies used were the mouse anti-IBA-1 microglia marker (1: 1000, SySy, 234,011), the rat anti-CD68 microglia activation marker (1: 1000, BioRad, MCA1957), the rabbit anti-Ki67 proliferation marker (1: 500, Thermofisher, MA5-14520), and the rabbit anti-COX4 inner mitochondrial membrane marker (1: 500, SySy, 298,002). Cells were washed 3 times and blocked again with 1% BSA for 5 min at room temperature on a shaker. They were then incubated with Cy2-AffiniPure Goat anti-Mouse IgG (H + L) (115–225-146), Cy5-AffiniPure Goat anti-Rat IgG (H + L) (112–175-167), and Cy3-AffiniPure Goat anti-Rabbit IgG (H + L) (111–165-144) secondary antibodies (1: 500, Jackson ImmunoResearch Laboratories) for 30 min at 30°C. Finally, nuclear DNA was counterstained with 1: 1000 DAPI for 5 min at room temperature and washed once with milliQ water for 5 min. The coverslips were then mounted on glass slides in fluorogel embedding medium (Electron Microscopy Sciences, Hatfield, PA).

### 2.5 Fluorescence microscopy

Immunostained images of single-cell microglia were acquired with the SIM technique using the ApoTome microscope (Axiocam 705 Imager.M2, Zeiss; Jena, Germany). Multiple optical z-sections of each cell were acquired at 0.25 μm intervals using the 63X oil immersion objective and deconvoluted using the ApoTome image function. Fluorescence intensities were analyzed using ImageJ (Fiji, version 2.9.0). For this purpose, all images in all tested groups were acquired with the same fluorescent light exposure time for each channel (IBA-1, CD68, and Ki67). Then, the microglial cells were selected as ROIs, and the integrated density of each ROI was measured. In each image, the mean gray level of three regions that did not exhibit fluorescence was evaluated as background. Then, the corrected total cell fluorescence was measured as (CTCF) = integrated density – (area of selected cell x mean fluorescence of background values). The data were normalized to the control group. For immunohistochemistry experiments, all slides were coded and analysis was performed blindly.

### 2.6 Morphological analysis

Changes in microglial morphology were analyzed as previously described (Fernández-Arjona et al., 2017). Images were first pre-processed by converting the green channel (IBA-1) to 8-bit grayscale images and then binarized to obtain black and white images by applying a selected auto-threshold (huang dark) in ImageJ. Then, the images were manually processed to remove pixels belonging to neighboring cells or artifacts in the background and add some pixels to join processes belonging to the selected cell. This step was performed by comparing the binary images with the original image. The branching data were obtained using the MorphData macro (Campos, 2021), which utilizes the AnalyzeSkeleton plugin (Arganda-Carreras et al., 2010). The different morphological parameters (cell perimeter, convex hull perimeter, cell circularity, convex hull circularity, cell area, convex hull area, fractal dimension, lacunarity, density, and roughness, convex hull span ratio, diameter of the bounding circle, maximum span across the convex hull, ratio maximum/minimum convex hull radii, mean radius) were analyzed using the FracLac plugin (Karperien, 2013). Next, the data from individual cells were combined into single summary files using the provided code of Campos et al. in Python (version 3.9.13). In the next step, the cell branching complexity was determined with Sholl Analysis plugin in ImageJ (Ferreira et al., 2014). Finally, a three-component principal component analysis (PCA) was performed by first scaling the data using the preprocessing. Scale function of the Sklearn library and then using the functions of the same library to fit the scaled data to obtain the coordinate data for the PCA graphs and further analysis (Sklearn version 1.0.2). The optimal number of clusters was selected using the elbow method, followed by K-Means clustering (Sklearn version 1.0.2) to select cells according to the degree of dissimilarity of morphological parameters (fractal dimension, lacunarity, density, span ratio major_minor, area of convex hull, convex hull perimeter, convex hull circularity, diameter of bounding circle, average radius, maximum span of convex hull, max_min radii, area of cell, perimeter of cell, roughness, circularity of cell, number of branches, average length of branches, maximum length of branches) to be clustered by dimensionality reduction. The matplotlib library was used to display the obtained data (matplotlib version 3.5.2). In addition, the numpy (version 1.21.5) and pandas (version 1.4.4) libraries were used for data processing.

### 2.7 Enzyme-linked immunosorbent assay (ELISA)

Cell supernatants were collected 24 h after treatment with poly I:C and UA and stored at −20°C until use. Mouse TNF-α DuoSet (DY410), mouse IL-1β/IL-1F2 DuoSet (DY401), mouse IL-10 DuoSet (DY417), and mouse CCL2/JE/MCP-1 DuoSet (DY479) ELISA kits (R&D Systems) were used to determine the levels of cytokines (TNF-α, IL-1β, IL-10) or Chemokines (CCL2) in the cell supernatants. In brief, 100 μL of cytokine-specific capture antibodies were applied to medium binding 96-well microplates and incubated overnight at room temperature. Plates were washed three times with 0.05% v/v Tween 20 in 1X PBS wash buffer (R&D Systems, Cat. WA126) and blocked in 300 μL 1% BSA in PBS reagent dilution (R&D Systems, Cat. DY995) for 1 h at room temperature. After washing again, 100 μL of the samples and standards were added and incubated for 2 h at room temperature. Plates were then washed three times and incubated with 100 μL of detection antibodies for 2 h at room temperature. The plates were rinsed and 100 μL of streptavidin-HRP was added per well and incubated for 20 min at room temperature in the dark. After rinsing again, the substrate solution [1: 1 A (H_2_O_2_) + B (tetramethylbenzidine), R&D Systems, cat. DY999] was added and incubated for 20 min, followed by 50 μL of the stop solution (R&D Systems, cat. DY994). The plates were read at 450 nm in an Epoch microplate reader using Gen5 software (BioTek, United States). Finally, the measured optical density of the reaction was compared with the optical density of the known standard samples to determine the protein concentration in the samples.

### 2.8 ROS detection assay

The production of reactive oxygen species, including peroxides, superoxides, hydroxyl radicals, and singlet oxygen, by treated cells was determined using a cellular ROS kit (Cat: ab113851; Abcam, Cambridge, United Kingdom) according to the manufacturer’s instructions. Briefly, 7–9 × 10^3^ microglia DIV 14 were plated with a primary embryonic hippocampal culture containing 2 × 10^4^ cells per well at DIV 21 in a 96-well plate. 48 h after microglia plating, cells were treated with poly I:C and UA for 24 h as described previously, but now in 1X HBSS medium without phenol red (giving a high autofluorescence for the ROS assay). Cells were then treated with 20 μM 2′,7′-dichlorodihydrofluorescein diacetate (H2DCFDA) for 30 min at 37°C and fluorescence was immediately measured in an Epoch microplate reader at 485 nm. H2DCFDA is deacetylated by cellular estarases to 2′,7′-dichlorodihydrofluorescein (H2DCF) and then oxidized by cellular ROS to 2′,7′-di chlorofluorescein (DCF). Treatment with tert-butyl hydroperoxide (TBHP) for 4 h served as a positive control for ROS production.

### 2.9 Statistical analysis

Data analysis was performed using Graphpad Prism 9 (Graphpad Software, Inc., United States). Data were presented as mean ± SEM. Differences between experimental groups were determined by one-way ANOVA for analyses with a single variable or by two-way ANOVA for analyses with two variables, followed by Fisher’s LSD *post hoc* test for multiple comparisons. The minimum significance value was considered as *p* < 0.05. The “n” of the different experimental groups is indicated in the respective figure legends. Since this study required the individual cells for analysis, some cultures did not yield up to 10 separate cells per coverslip, as cells overlapping other cells were excluded from imaging to avoid analyzing cumulative fluorescence from more than one cell. In addition, some cells were not of sufficient quality to be reliably analyzed by the software. For these reasons, the number of cells in the figures may vary. All experiments were evaluated in a strictly blind fashion.

## 3 Results

### 3.1 Urolithin A attenuates microglial reactivity triggered by poly I:C stimulation

**Figure 2:**
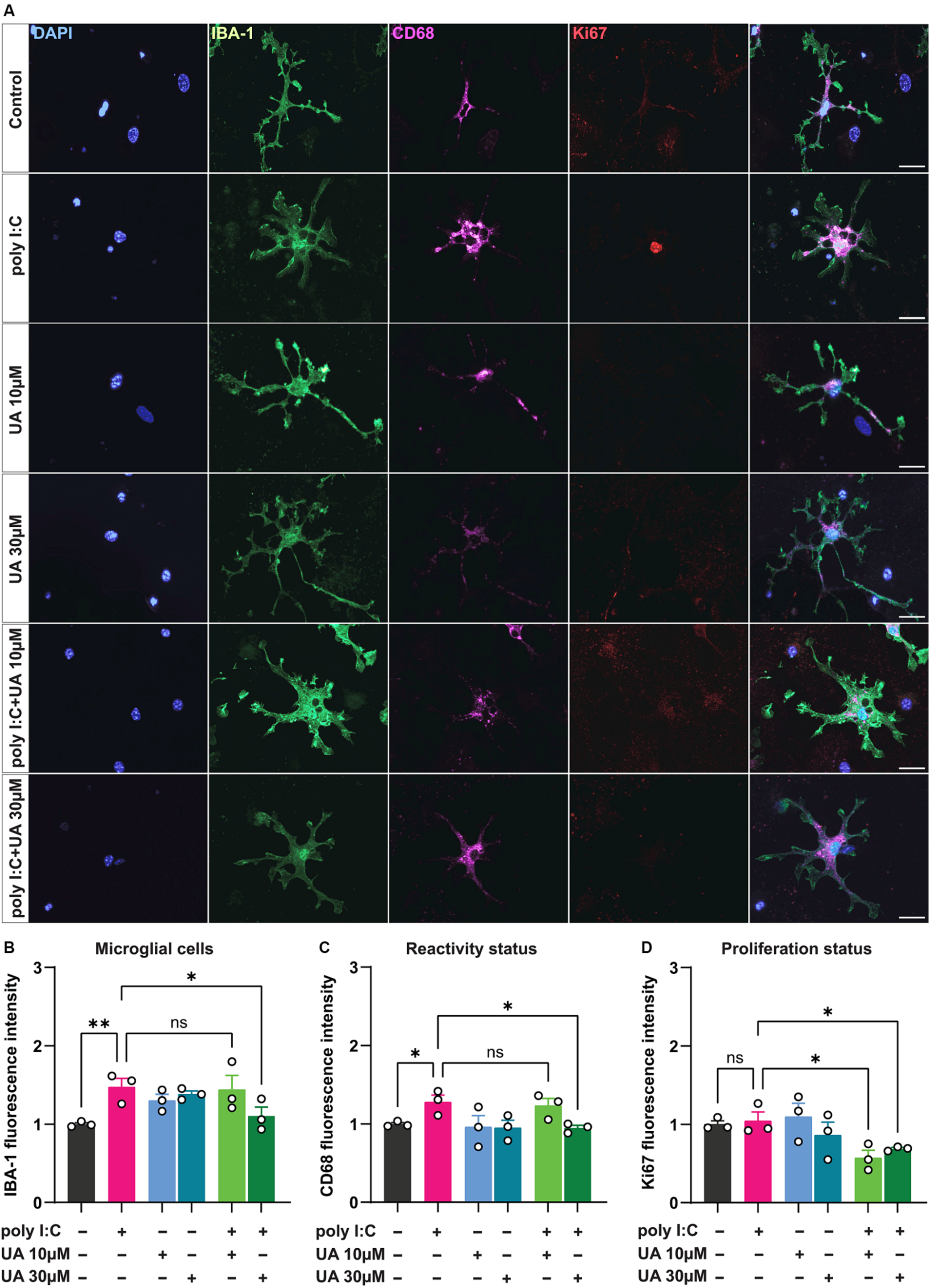
Urolithin A reduced the microglia reactivity induced by poly I:C. Triple co-cultures were treated with 50 μg/mL poly I:C alone or together with either 10 or 30 μM UA for 24 h, followed by immunocytochemistry for IBA-1, CD68 and Ki67. Single cell images of microglia were captured using an ApoTome microscope (63× objective). **(A)** Representative immunofluorescence images show the microglia activation markers IBA-1 (green) and CD68 (magenta) and the proliferation marker Ki67 (red). Scale bar: 20 μm. **(B-C)** Relative change in corrected total cell fluorescence intensity of IBA-1 and CD68, indicating the degree of microglia reactivity in the different experimental groups. Number of experiments, *N* = 3, *n* = 16–31 cells per group. **(D)** Relative change in fluorescence intensity of Ki67 indicating cell proliferation in the different experimental groups. *N* = 3, *n* = 12–22 cells per group. Data are presented as mean ± SEM and were analyzed by one-way ANOVA followed by Fisher’s LSD test; *p < 0.05 and **p < 0.01.

Under pathological conditions, microglial cells proliferate and adopt a reactive status to respond to the pathogenic insult (Streit et al., 1999; Sahu and Ter, 2018; Li et al., 2019; Edler et al., 2021). To evaluate the microglial reactivity triggered by the viral mimetic poly I:C and the potential beneficial effect of UA, triple co-cultures were treated simultaneously or alone with poly I:C and UA (10 and 30 μM). For this purpose, immunostaining was performed for IBA-1 and CD68, which are known to be upregulated in reactive microglial cells (Lier et al., 2021). The results showed that poly I:C-induced immunostimulation significantly increased the expression levels of the microglial reactivity-related proteins IBA-1 (*p* = 0.007) and CD68 (*p* = 0.04) compared to control (Figures 2A–C). The expression levels of both proteins related to microglial reactivity were significantly reduced when cultures were treated simultaneously with poly I:C and 30 μM UA compared to cultures treated with poly I:C alone (IBA-1: *p* = 0.027, CD68: *p* = 0.022). However, lower concentrations of UA (10 μM) did not significantly alter the increased IBA-1 and CD68 levels in microglial cells induced by poly I:C (F_IBA-1_ (5, 12) = 3.38, *p* = 0.03; F_CD68_ (5, 12) = 2.90, *p* = 0.06; Figures 2A–C).

Microglial cells have been shown to proliferate when exposed to danger signals such as pathogens, protein aggregates, pro-inflammatory cytokines, injury, and cellular stress (He et al., 2021). To further investigate the potential beneficial role of UA in preventing poly I:C-induced microglial proliferation, treated cultures were immunostained for the nuclear proliferation marker Ki67 in addition to IBA-1. The results showed that poly I:C administration did not result in a significant increase in Ki67 levels in microglial cells compared with control group (*p* = 0.79). Interestingly, however, simultaneous treatment of co-cultures with poly I:C and either 10 or 30 μM concentrations of UA resulted in a significant decrease in Ki67 expression levels in microglial cells compared to poly I:C administration alone (UA 10 μM: *p* = 0.013, UA 30 μM: *p* = 0.049; F_Ki67_ (5, 12) = 3.31, *p* = 0.04; Figure 2D).

### 3.2 Urolithin A reverses poly I:C-induced morphological changes in microglial cells

**Figure 3:**
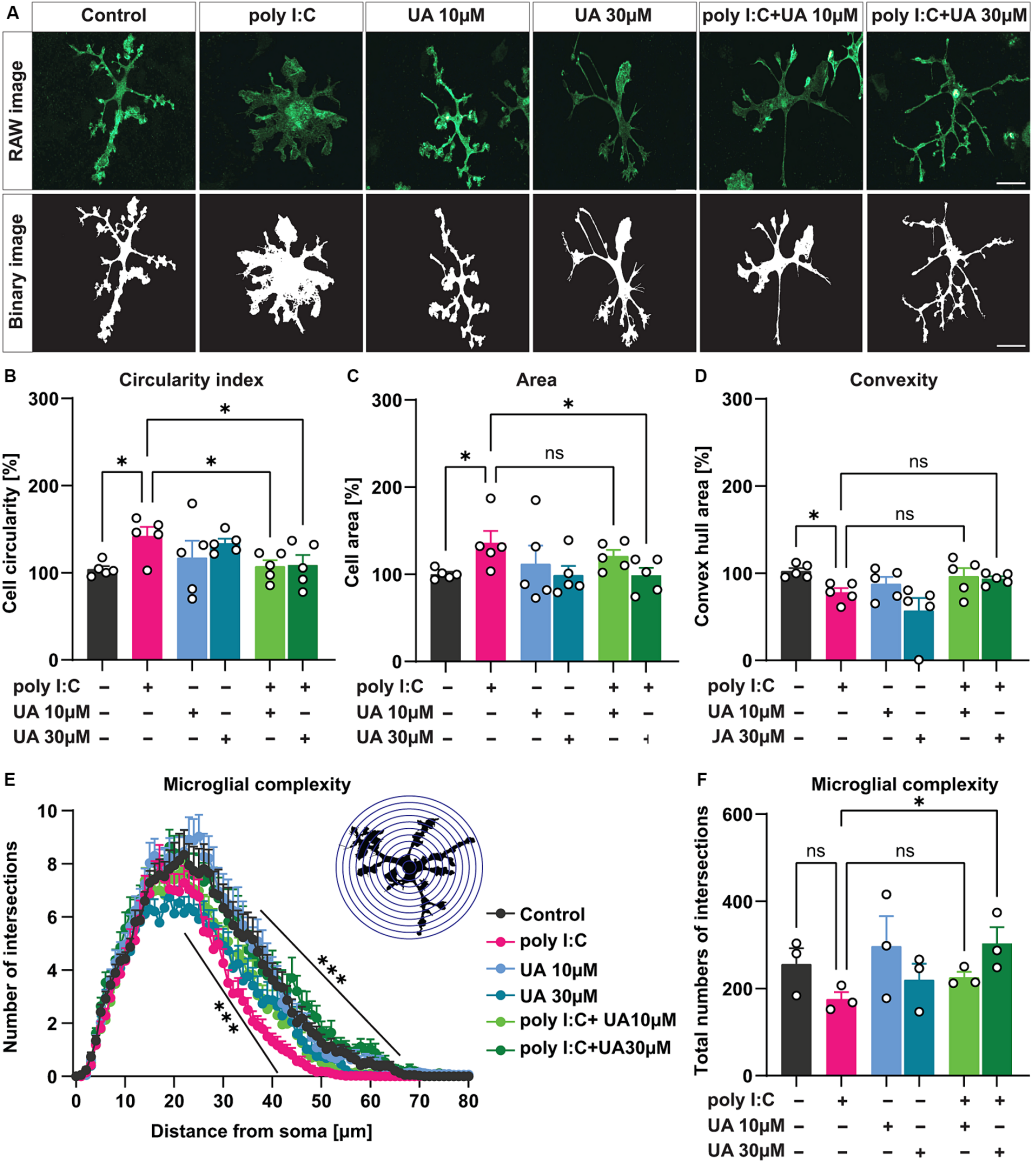
Urolithin A rescued poly I:C-induced morphological changes in microglial cells. Microglial cells were immunostained with IBA-1 and morphological features were analyzed with MorphData and the FracLac plugin. **(A)** Representative immunofluorescence images showing the microglia marker IBA-1 and corresponding binary images (black and white) for morphological analysis. Scale bar: 20 μm. **(B–D)** Morphometric parameters such as circular shape, cell area and convex hull area showing changes in microglia morphology were shown. *N* = 5, *n* = 40–66 cells per group. Data are presented as mean ± SEM and were analyzed by one-way ANOVA followed by Fisher’s LSD test. **(E)** Sholl analysis showing the complexity of microglial branching in terms of the number of process crossings and distance from the cell soma in 1 μm increasing radius of concentric circles. *N* = 3, *n* = 33–42 cells per group. Data are presented as mean ± SEM and were analyzed using two-way ANOVA with repeated measures followed by Fisher’s LSD test. **(F)** The total number of branching of microglial cells from the cell soma is shown. Data are presented as mean ± SEM and were analyzed by one-way ANOVA followed by Fisher’s LSD test; *p < 0.05, **p < 0.01 and ***p < 0.001.

In a pathological state, microglia can undergo a characteristic morphological change from a branched, ramified state to an intermediate, reactive phenotype and eventually to an amoeboid shape (Streit et al., 1999; Sahu and Ter, 2018; Li et al., 2019; Edler et al., 2021). To determine the potential role of UA in preventing this activation-induced change in microglial morphology induced by poly I:C, the morphological features of individual microglial cells were analyzed by ImageJ using the MorphData macro and FracLac plugin. For this analysis, the IBA-1-positive cells in each experimental group were randomly selected, and the corresponding binary images were generated using ImageJ and subsequently analyzed (Figure 3A). The results showed that simultaneous treatment of the cultures with poly I:C and UA resulted in a significant concentration-dependent effect (UA 10 μM: *p* = 0.032, UA 30 μM: *p* = 0.041) that suppressed the poly I:C-induced increase in the circularity index of the cells (*p* = 0.02), the characteristic form of reactive amoeboid microglial cells (F_Circularity_ (5, 24) = 2.07, *p* = 0.01; Figure 3B). Moreover, treatment with 30 μM UA was able to reduce the increase in microglial cell area induced by poly I:C (*p* = 0.039; F_Area_ (5, 24) = 1.55, *p* = 0.21; Figure 3C). In addition, UA treatments showed a trend of increasing convex hull area, which was significantly reduced in the poly I:C treated group (*p* = 0.046; F_Convexity_ (5, 24) = 3.98, *p* = 0.009; Figure 3D).

Next, Sholl analysis was performed to determine the branching complexity of microglial cells in the different experimental groups. Strikingly, the results showed that administration of poly I:C resulted in a significant decrease in the branching complexity of microglial cells, which may indicate that microglial cells adopt a more intermediate or amoeboid phenotype after stimulation with poly I:C, confirming their reactivity. However, simultaneous treatment of cultures with poly I:C and UA prevented the poly I:C-induced decrease in branching complexity of microglial cells, which was more pronounced at higher UA concentrations [F_Complexity_ (5, 219) = 4.35, *p* = 0.0008; Figure 3E]. Moreover, the total number of branched junctions of microglial cells tended to be reduced after administration of poly I:C, which was significantly restored in the presence of a higher concentration of UA [*p* = 0.04, F_Complexity_ (5, 12) = 1.55, *p* = 0.24; Figure 3F].

**Figure 4:**
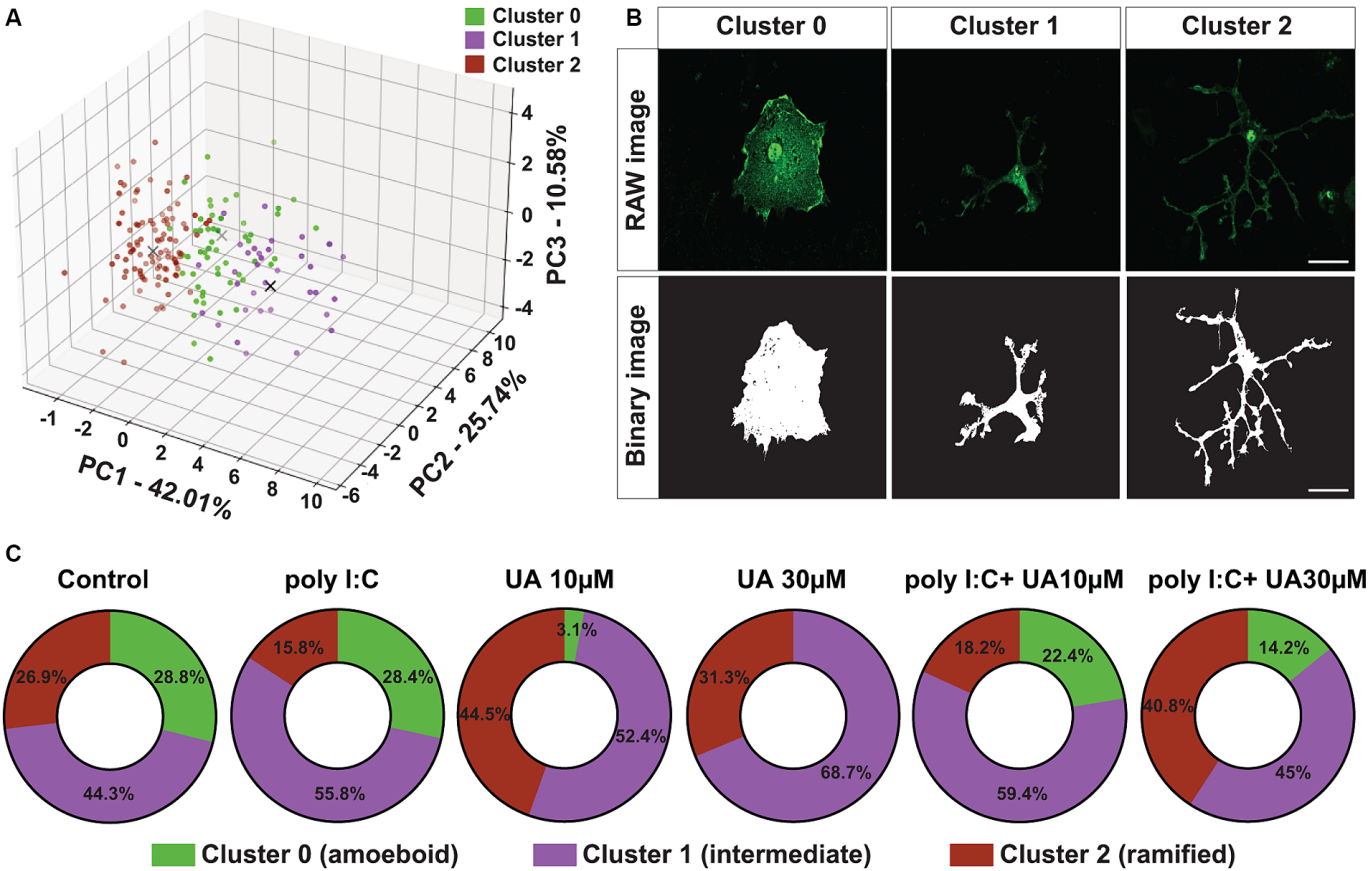
Urolithin A reversed the poly I:C-induced shift in microglia morphology. **(A)** Principal component analysis by dimensionality reduction and K-Means clustering of microglial cells into three different clusters based on the degree of dissimilarity of their morphological parameters are shown. **(B)** Representative immunofluorescence images show IBA-1-positive microglia (green) and corresponding binary images (black and white). Scale bar: 20 μm. **(C)** Quantification of PCA and K-Means data showing the percentage (%) changes of the different clusters between the experimental groups.

To further investigate the morphological change of microglial cells induced by poly I:C stimulation and the possible positive role of UA, a principal component analysis (PCA) was performed. This analysis was carried out with three principal components to visualize and combine the morphological features of microglial cells in the different experimental groups. The results showed that principal component 1 (PC1) explained 42.01% of the variation in the data, PC2 explained 25.74% variation and PC3 explained 10.58% variation (Figure 4A). To analyze the different clusters of cells exhibiting different morphological phenotypes, K-Means clustering was performed on the PCA data. The optimal number of clusters was selected using the elbow method. PCA clustering resulted in three different clusters, 0, 1 and 2, which were identified as amoeboid, intermediate and ramified cells, respectively (Figure 4B).

Compared to the control group, poly I:C stimulation resulted in an 11.1% decrease in the ramified cluster of microglial cells and an 11.5% increase in intermediate cluster. However, in the first cluster, which contained amoeboid microglial cells, only minor changes were observed between the control group and the poly I:C-treated cultures (Figure 4C). Simultaneous treatment of the cultures with poly I:C and UA reversed this poly I:C-induced morphometric shift of the microglial cells in a concentration-dependent manner. Remarkably, treatment with 30 μM UA in combination with poly I:C increased the ramified cluster of microglial cells by 25%, reduced the intermediate cluster by 10.8% and reduced the amoeboid cluster of microglial cells by 14.2%. The lower concentration of UA (10 μM) did not lead to a significant shift in microglial morphology (Figure 4C).

Interestingly, treatment of the cultures with UA alone also led to an increase in the population of ramified and intermediate cells and to a massive reduction or even absence of populations of ameobid microglial cells (Figure 4C). These results suggest that UA can not only prevent the poly I:C-induced morphological shift of microglial cells from a branched to an intermediate phenotype, but that UA *per se* even has positive effects on the shape of microglial cells in culture, helping them to adopt a ramified status.

### 3.3 Urolithin A reduces the level of secreted pro-inflammatory mediators induced by poly I:C in the culture supernatant

Pro-inflammatory cytokines and chemokines are molecules secreted by peripheral immune cells and by microglia, astrocytes, and neurons in the CNS (Galic et al., 2012). The production of pro-inflammatory mediators in the brain is one of the most important mechanisms that develop in many pathological conditions. Although they can be helpful in limiting the stimulus, their higher concentration and prolonged secretion can have negative effects on neuronal cells (Hosking and Lane, 2010; DiSabato et al., 2016). Previously, it has been shown that immune stimulation by viral or bacterial insults can trigger the production of pro-inflammatory mediators in the CNS or in cell cultures (Jang et al., 2012; Hosseini et al., 2018; He et al., 2021). On the other hand, several studies have shown that UA is able to limit inflammatory signaling under various pathological conditions (D'Amico et al., 2021). Therefore, the anti-inflammatory properties of UA were analyzed here in terms of a possible reduction of pro-inflammatory mediators secreted into the culture supernatant after poly I:C administration.

**Figure 5:**
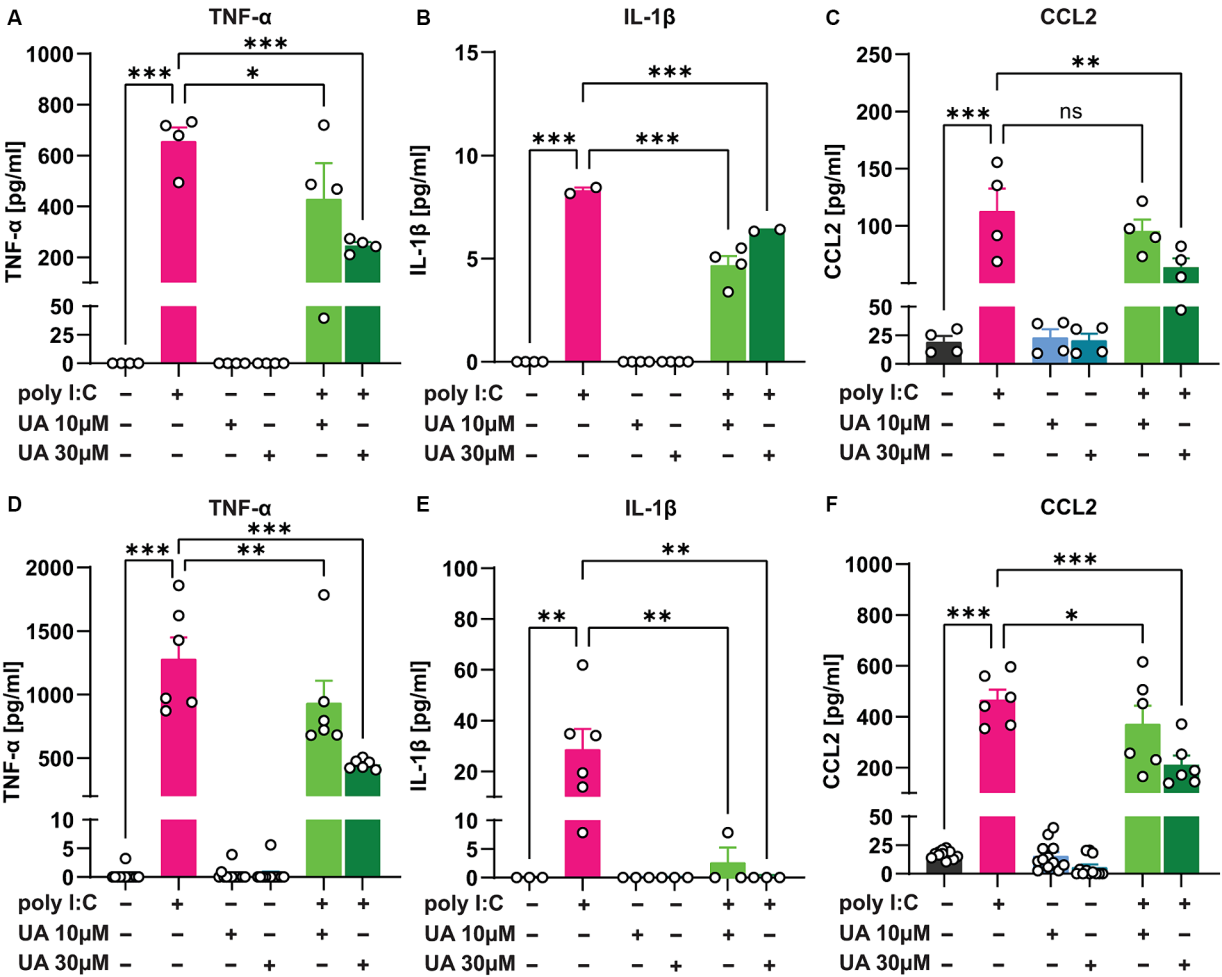
Urolithin A reduced the release of pro-inflammatory mediators induced by poly I:C in the culture supernatant. Either the triple co-cultures or the primary pure microglia cultures were treated with poly I:C (50 μg/mL) and UA (10 μM & 30 μM) for 24 h, and the pro-inflammatory mediators were analyzed in the supernatant by ELISA. **(A–C)** Shown are the protein concentrations of the pro-inflammatory cytokines TNF-α, IL-1β and the chemokine CCL2 in the supernatant of triple co-cultures and **(D–F)** microglia monocultures. *N* = 3–12. Data are presented as mean ± SEM and were analyzed by one-way ANOVA followed by Fisher’s LSD test; *p < 0.05, **p < 0.01 and ***p < 0.001.

The results showed that 24 h after administration of poly I:C in the triple co-culture, the levels of the pro-inflammatory cytokines TNF-α (*p* < 0.001, Figure 5A) and IL-1β (*p* < 0.001, Figure 5B) as well as the chemokine CCL2 (*p* < 0.001, Figure 5C) were significantly increased in the supernatant of the culture. However, simultaneous treatment of the triple co-cultures with UA and poly I:C reduced the levels of these pro-inflammatory mediators, especially at higher concentrations of UA (TNF-α: *p* = 0.0002, Figure 5A; IL-1β: *p* = 0.0005, Figure 5B; CCL2: *p* = 0.004, Figure 5C). Therefore, treatment with UA at higher concentrations was able to significantly reduce the amount of pro-inflammatory mediators secreted in the supernatant of triple co-cultures stimulated with poly I:C [F_TNF-α_ (5, 18) = 19.53, *p* < 0.0001; F_IL-1β_ (5, 14) = 208.5, *p* < 0.0001; F_CCL2_ (5, 18) = 15.32, *p* < 0.0001; Figures 5A–C]. To further investigate whether UA exerts its positive effect in reducing the secretion of pro-inflammatory mediators in the triple co-culture by influencing microglial cells, the pure primary microglial cultures were stimulated with poly I:C in further experiments and the effects of UA on the reduction of pro-inflammatory mediators were analyzed (Figures 5D–F). The results showed that in pure primary microglia cultures, stimulation with poly I:C led to a significant increase in TNF-α (*p* < 0.001, Figure 5D), IL-1β (*p* = 0.002, Figure 5E) and CCL2 levels (*p* < 0.001, Figure 5F) in the supernatant, even to a greater extent than in the triple co-culture. And simultaneous treatment of the cultures with UA resulted in a significant decrease in these pro-inflammatory mediators (TNF-α: *p* < 0.001, Figure 5D; IL-1β: *p* = 0.002, Figure 5E; CCL2: *p* < 0.001, Figure 5F). Thus, treatment with UA was also shown to significantly affect microglial function and reduce the production of pro-inflammatory mediators induced by poly I:C [F_TNF-α_ (5, 48) = 62.59, *p* < 0.001; F_IL-1β_ (5, 15) = 5.24, *p* = 0.005; F_CCL2_ (5, 48) = 59.65, *p* < 0.0001; Figures 5D–F].

### 3.4 Urolithin A may induce mitochondrial biogenesis and attenuate poly I:C-induced ROS production

**Figure 6:**
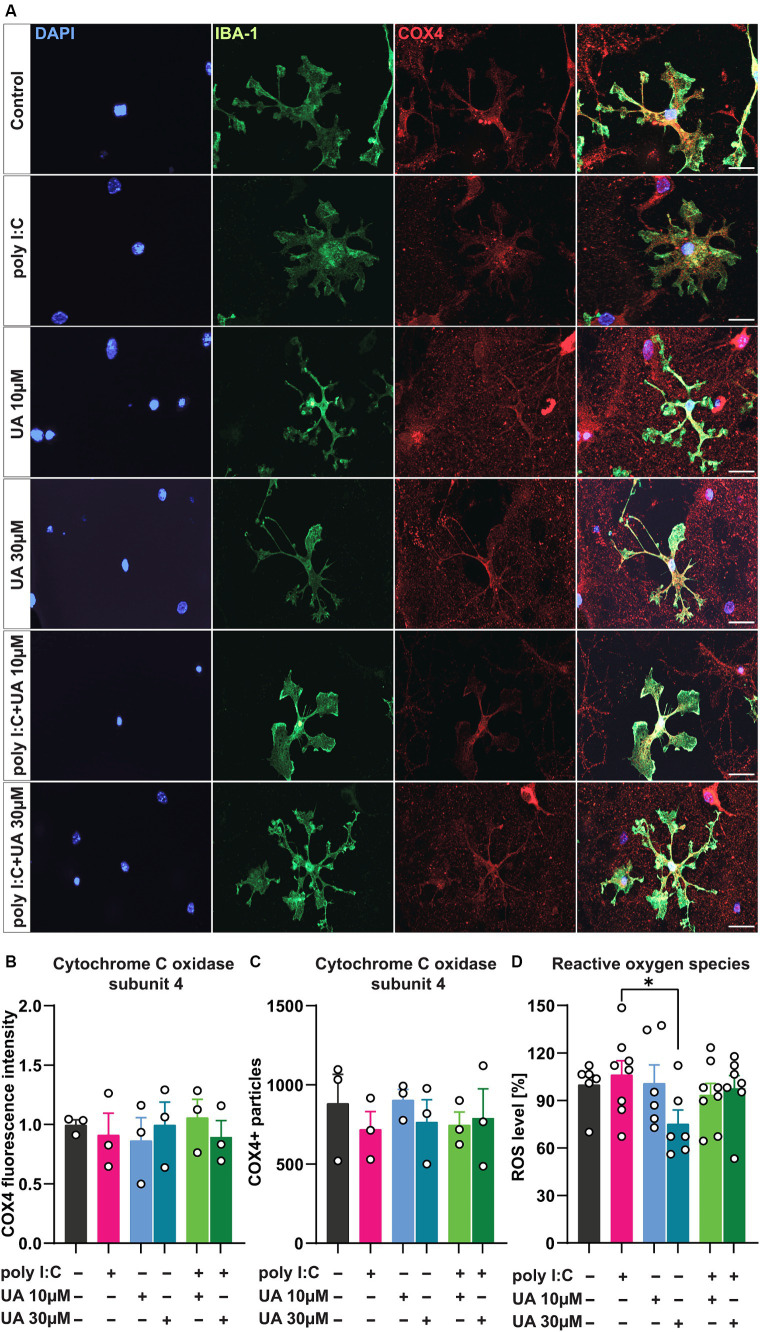
Urolithin A may increase mitochondrial biogenesis in microglial cells and limit cellular ROS production induced by poly I:C. Cultures were treated with 50 μg/mL poly I:C and UA (10 μM & 30 μM) for 24 h and analyzed immunocytochemically for the internal mitochondrial protein cytochrome C oxidase (COX4). For ROS measurements, cells were incubated with H2DCFDA for 30 min and the colorimetric assay was perfected. **(A)** Representative immunofluorescence images show microglia, IBA-1 (green), and mitochondria, COX4 (red). Scale bar: 20 μm. **(B)** Relative COX4 fluorescence intensity and **(C)** number of COX4+ particles in microglial cells of the different experimental groups. *N* = 3, *n* = 20–34 cells per group. **(D)** Relative ROS content - the amount of DCF generated upon oxidation of H2DCF by cellular reactive oxygen species in the different groups is shown. *N* = 6–8. Data are presented as mean ± SEM and were analyzed by one-way ANOVA followed by Fisher’s LSD test; *p < 0.05.

Viral infection has been shown to trigger microglial cell activation, which is associated with mitochondrial dysfunction and excessive production of ROS (Thangaraj et al., 2018; Eshraghi et al., 2021). In addition, some studies suggest that UA can improve mitochondrial function under various pathological conditions by stimulating mitophagy, a process in which damaged mitochondria are recycled to allow renewal by healthy mitochondria (Ryu et al., 2016). Under pathological conditions, UA was also able to reduce the production of ROS in cells and inhibit the apoptosis pathway associated with mitochondria (Qiu et al., 2022). To investigate the role of UA in preventing poly I:C-induced mitochondrial dysfunction in microglial cells in this scenario, the expression level of the inner mitochondrial protein cytochrome C oxidase type 4 (COX4), the regulator of oxidative phosphorylation, was first quantified in the microglial cells of the different experimental groups (Figure 6A). Although the assessment of COX4 fluorescence intensity (Figure 6B) as well as the number of COX4-positive particles (Figure 6C) showed no significant differences between the experimental groups, the administration of poly I:C tended to decrease COX4 levels, which was slightly reversed in the groups treated simultaneously with poly I:C and UA [F_COX4 FL intensity_ (5, 12) = 0.21, *p* = 0.94; F_COX4 particles_ (5, 12) = 0.30, *p* = 0.89; Figures 6B,C].

To test the possible positive role of UA in preventing the release of ROS, indicating a reduction of oxidative stress in the cultures after poly I:C stimulation, the next step was to determine the ROS content in the supernatant of the triple co-cultures using a colorimetric assay (Figure 6D). The results showed that the administration of a higher concentration of UA (30 μM) alone led to a decrease in the ROS content in the supernatant of the cultures (*p* < 0.05), which may parallel the effects of UA *per se* in increasing the populations of ramified microglial cells in the cultures. However, only a slight increase in ROS concentration was observed in the supernatant of the cultures induced by poly I:C, which was slightly lower when the cultures were treated with poly I:C and UA at the same time [F_ROS_ (5, 35) = 1.49, *p* = 0.22; Figure 6D].

## 4 Discussion

Viral infections still represent a major threat to human health today. Although many of the viruses have a specific target tissue, neurotropic viruses have evolved mechanisms to exploit weaknesses in immune defenses that eventually allow them to reach and infect cells of the central nervous system (CNS) (Hosseini and Korte, 2023). Once in the CNS, these viruses can cause severe neuronal damage, sometimes with long-lasting, life-threatening consequences. Remarkably, even the ability to enter the CNS and cause neuronal infection does not appear to be a determining factor in whether a viral strain causes neurological complications (Hosseini et al., 2018; Jurga et al., 2020). To date, viral infections are discussed as a risk factor for the development or progression of neurodegenerative diseases (Klein et al., 2019). The cellular mechanisms underlying the neurological consequences of viral infections are not fully understood, but they involve neuroimmune interactions that have so far mainely focused on microglia. As the major immune cells in the brain, reactive microglia play a central role in neuroinflammation by responding directly or indirectly to viruses. Chronic reactivity of microglia, characterized by alteration of microglial morphology, proliferation and release of neurotoxic molecules, leads to functions that differ from their beneficial role under physiological conditions and may result in neuronal damage contributing to the pathogenesis of various neurological diseases (Jang et al., 2012; Sadasivan et al., 2015). As the CNS is highly susceptible to inflammatory reactions and has a limited regenerative capacity (Chesnokova et al., 2016), disruptions to its homeostasis can have profound consequences for important functions and severely affect patients’ quality of life. Therefore, it is of great importance to find an effective way to prevent harmful neurological consequences of viral infections of the brain. To investigate the potential beneficial effects of urolithin A (UA), a metabolite of intestinal bacteria that has shown anti-inflammatory effects in various pathological conditions (Ryu et al., 2016; Gong et al., 2019; D'Amico et al., 2021; Qiu et al., 2022), on the prevention of neuroinflammation triggered by viral infections, a triple co-culture system containing all three major types of brain cells - neurons, astrocytes and microglia - was used. The viral mimetic used was a synthetic analog of viral dsRNA, poly I:C, which is known to induce microglial activation and inflammatory responses (Woodburn et al., 2021). The results indicated that UA significantly prevented microglial reactivity and rescued the phenotypic shift from ramified cells to reactive intermediate cells after poly I:C stimulation. In addition, UA significantly decreased the levels of pro-inflammatory mediators (TNF-α, IL-1β and CCL2) and a trend to decrease ROS levels released in the supernatant of poly I:C-treated cultures.

The results of this study showed that the simultaneous administration of UA at a higher concentration (30 μM) and poly I:C in cultures resulted in reduced expression of IBA-1 and CD68 in microglial cells. For the interpretation of these results, it is important to note that IBA-1 only labels microglia/infiltrating macrophages and that its expression level is related to the activation of microglia in brain tissue, as shown in recent studies (Jurga et al., 2020). On the other hand, a high expression level of CD68 in microglia is indicative of microglial cell reactivity. Consistent with these results, the positive role of UA in attenuating microglial reactivity in primary microglia cultures after stimulation with other pathogenic molecules such as lipopolysaccharide (LPS) has also been demonstrated (Xu et al., 2018; Velagapudi et al., 2019; Qiu et al., 2022). Although poly I:C resulted in microglial cell reactivity that was attenuated by UA, it did not significantly increase microglial cell proliferation as assessed by Ki67 expression in the cells. This is consistent with the observation of He et al. who showed that poly I:C did not induce significant proliferation of primary microglial cells (He et al., 2021). Nevertheless, the low proliferation of microglial cells after stimulation with 50 μg/mL poly I:C may indicate that long-term stimulation or higher concentrations are required to achieve stronger effects. Indeed, viral infection is progressive, and the degree of microglial activation depends on the concentration of the immunogenic molecule (Sahu and Ter, 2018; Woodburn et al., 2021). It is also plausible that the complex interactions between microglia, neurons and astrocytes in the triple co-culture used here limited the extent of microglial proliferation compared to monocultures of microglia. However, Ki67 expression in microglial cells was also lower after simultaneous treatment with poly I:C and UA, again confirming the positive role of UA under these experimental conditions. Since UA can cross the blood–brain barrier (Gasperotti et al., 2015), this result may suggest that UA as a therapeutic agent after viral infections may be able to limit microglial activation and proliferation, which remains to be investigated.

In addition to a higher expression of activation-related markers in microglial cells triggered by poly I:C, this stimulus led to a shift in microglial morphology from small branched cells to larger and rounder cells. Consistent with previous studies, an increase in cell circularity, an increase in cell area, and a decrease in convex hull area were observed as signs of microglial reactivity after poly I:C administration (Fernández-Arjona et al., 2017; Leyh et al., 2021). Remarkably, UA was able to reduce the poly I:C-induced increase in cell circularity and cell area that define the characteristic intermediate and amoeboid shape of activated microglial cells. In addition, there was a trend toward an increase in the convex hull area of microglial cells, indicating an increase in the expansion of cell extensions, as microglial cell areas tended to be smaller in cultures treated with poly I:C that were simultaneously treated with UA.

To date, the role of UA in modulating immunostimulant-induced microglial activation, particularly in the morphological changes, has not been recognized. PCA analysis showed that stimulation with poly I:C shifted microglia to the intermediate activated phenotype rather than that of amoeboid cells. This is consistent with the findings of He et al. who found that in contrast to LPS, which induces an amoeboid morphology, poly I:C mainly induces an intermediate/activated morphology (He et al., 2021). Here, UA not only rescued poly I:C induction of intermediate and reduction of ramified microglia, but also significantly reduced the number of amoeboid microglial cells. This result suggests that UA treatment reverts microglia to their ramified state, with reduced circularity of cells, reduced cell area and increased branching. This branching phenotype of microglial cells is important for performing useful functions such as immune surveillance (Nimmerjahn et al., 2005). UA can therefore restore the homeostatic functions of microglia after immunostimulation. Fernandez-Arjona et al. have previously demonstrated *in vivo* aberrations in the morphology of microglia after administration of the surface protein of influenza viruses, neuraminidase, to rats (Fernández-Arjona et al., 2017).

It is likely that the morphological changes in microglial cells *in vivo* as a result of treatment with UA indicate a possible prevention of neuroinflammatory processes. On the other hand, as described by Woodburn et al., it should be kept in mind that morphology does not always define the functional state of microglial cells (Woodburn et al., 2021). Therefore, the observed restoration of microglia morphology by UA treatment should not be interpreted as a linear change in their functions and requires further detailed investigation and the analysis of biomarkers for microglial activation. But overall, the results of this study regarding the reduction of IBA-1 and CD68 and the changes in microglia morphology toward a reduced activation status, in line with previous observations of the anti-inflammatory role of UA in various pathological conditions, suggest a potentially positive role of UA in virus-induced microglia activation.

To determine whether the rescue of poly I:C-induced morphological changes by UA can positively affect microglial functionality, the results showed that UA dramatically reduced the release of neurotoxic molecules in the microenvironment of cells 24 h after poly I:C stimulation in both the triple co-culture and the primary microglia monoculture. UA significantly reduced the release of pro-inflammatory cytokines (TNF-α and IL-1β) and chemokines (CCL2) after poly I:C treatment. This finding confirms that UA can exert anti-inflammatory functions in microglia during immune stimulation, which is consistent with several studies in other pathological conditions (Xu et al., 2018; DaSilva et al., 2019). DaSilva et al. (2019) have previously shown that the reduction of pro-inflammatory mediators in microglia can prevent neuronal apoptosis in microglia–neuron co-cultures (DaSilva et al., 2019). Furthermore, UA did not induce detectable changes in IL-10 levels 24 h after treatment (Data are not shown). This suggests that UA may only attenuate inflammation by suppressing pro-inflammatory mediators and does not stimulate the production of anti-inflammatory mediators. Future determinations of microglial gene profiles will certainly shed further light on these conclusions. However, Abdelazeem et al. (2021) found that UA increased IL-10 levels 2 h after LPS stimulation of bone marrow-derived macrophages, but decreased at later time points (Abdelazeem et al., 2021). Therefore, cytokine analysis at different time points or detection of mRNA will be useful to assess the role of UA in the release of IL-10 and other anti-and pro-inflammatory cytokines. Nonetheless, the decrease in pro-inflammatory molecules could have a significant physiological effect in limiting neuroinflammation and the resulting neuronal damage.

Over the past 10 years, evidence has accumulated that patients infected with RNA viruses are under chronic oxidative stress (Liu et al., 2017). Oxidative stress from RNA virus infection may contribute to various aspects of viral disease pathogenesis, including inflammatory responses, cell death, immune system dysregulation and enhanced viral replication (Reshi et al., 2014). Consequently, the accumulation of oxidative damage to mitochondrial DNA in microglia under pathological conditions, including viral infections, leads to increased production of ROS, which subsequently leads to neuronal damage and death (Kaul et al., 2001; Thangaraj et al., 2018).

Next, the role of UA in attenuating mitochondrial damage and oxidative stress, which have been shown to occur in viral infections, was investigated (Thangaraj et al., 2018; Foo et al., 2022; Pliss et al., 2022). In contrast to other studies showing that UA induces mitophagy by decreasing mitochondrial accumulation and thus the expression of mitochondrial proteins such as COX4, here UA instead led to a very slight increase in COX4 expression. Although this change was statistically insignificant, it could indicate an increase in mitochondrial biogenesis. This is similar to the observations of Esselun et al. who found that UA did not induce mitophagy, but did increase the transcription of genes for mitochondrial biogenesis (Esselun et al., 2021). Another study also found that UA triggers cellular autophagy rather than mitophagy in ischemic neuronal injury (Ahsan et al., 2019). While these results appear contradictory at first glance, they may represent observations at different stages of the same pathway. Indeed, an increase in mitophagy would degrade the accumulated damaged mitochondria in the short term and eventually increase mitochondrial biogenesis after recycling the materials. However, to determine the role of UA in preventing mitochondrial dysfunction in microglial cells triggered by an immune stimulus, a more robust analysis is required.

In addition, UA showed a slight trend to reduce cellular ROS production in cultures treated with poly I:C, although not statistically significant. This antioxidant property suggests that UA may limit oxidative stress in activated microglia (Qiu et al., 2022). In this case, the 8% reduction in ROS concentration induced by poly I:C following concomitant treatment with 30 μM UA could have a physiologically relevant effect in modulating neuroinflammation and maintaining brain homeostasis during viral infections. Specific quantification of mitochondrial ROS levels will provide a better idea of the role of UA in inhibiting ROS production. However, other studies suggest that the role of UA in improving mitochondrial function is independent of ROS production (Ryu et al., 2016; Esselun et al., 2021). Therefore, further studies are needed to objectively investigate the antioxidant role of UA in this scenario.

However, this study had the following limitations. First, this study primarily focused on elucidating the protective role of UA against viral mimetic-induced microglial dysfunction, as these cells play an important role in neuroinflammation and subsequent neuronal injury as resident innate immune cells of the CNS. However, recent data suggest that viruses and other immune stimuli can also activate astrocytes (Hosseini and Korte, 2023; Jorgačevski and Potokar, 2023). Thus, astrogliosis could also play an important role in neuroinflammation, which should be considered in future studies on therapeutic approaches. On the other hand, it is important to investigate the potential positive role of UA on poly I:C-induced neuronal death and damage to confirm that the role of UA in reducing microglial activation is sufficient to protect neurons in this scenario. Here, we used an optimized triple co-culture system comprising primary microglia, neurons and astrocytes to account for the complex interactions between these cells *in vivo* during neuroinflammatory processes. However, *in vitro* models cannot adequately represent the physiological state of the cells. Therefore, future studies in animal models and using real RNA virus infections will be useful to determine the role of UA *in vivo* in virus-induced neuroinflammation and to utilize it clinically in the long term. One area of microglial biology that has been relatively neglected until recently is sex differences. This is despite the fact that sex is a risk factor for several diseases characterized by neuroinflammation and thus also by microglial activation (Sumien et al., 2021). Therefore, sex differences should also be taken into account in further studies. Finally, this study does not shed light on the intracellular signaling mechanisms of UA in modulating microglial structure and function during viral stimulation, which warrants further investigation.

In conclusion, these results promote the notion that UA might be able to prevent or at least dampen microglial reactivity induced by viral poly I:C mimetic stimulation *in vitro*. UA was able to attenuate microglial reactivity by restoring the morphological changes of microglial cells and inhibiting the production of pro-inflammatory signaling molecules triggered by poly I:C stimulation. In addition, UA appears to promote mitochondrial biogenesis and suppress oxidative stress, which could prevent neuronal damage and death. Furthermore, the results of this study add detailed microglial morphometric findings at the single cell level to other studies showing the positive role of UA in preventing neuroinflammation under pathologic conditions. However, the molecular mechanisms underlying the protective effect of UA on microglial reactivity remain to be elucidated. In addition, the effect of UA on neurotoxicity mediated by reactive microglia and astrocytes should be considered. Therefore, the tripartite crosstalk and interactions between neurons, astrocytes and microglia cell types may provide a clearer picture of this scenario.

Since urolithins are natural metabolites of the gut microbiota from ellagitannins and ellagic acid and can be obtained from abundant foods such as pomegranates, strawberries and walnuts, they have promising therapeutic potential. Therefore, this study should be conducted *in vivo* and it will certainly contribute to the future use of UA in probiotics or as a dietary supplement to protect neurons from damage caused by neuroinflammation.

## Data availability statement

The original contributions presented in the study are included in the article/Supplementary material, further inquiries can be directed to the corresponding author.

## Ethics statement

All cell culture procedures have been reviewed and approved by the local committees of the TU Braunschweig, Germany, and the authorities (LAVES, Oldenburg, Germany) according to the national guidelines of the animal welfare law in Germany. The study was conducted in accordance with the local legislation and institutional requirements.

## Author contributions

YM: Data curation, Formal analysis, Investigation, Methodology, Software, Writing – original draft, Writing – review & editing. LG: Data curation, Formal analysis, Methodology, Software, Writing – review & editing. NL: Formal analysis, Investigation, Methodology, Writing – review & editing. BB: Supervision, Validation, Visualization, Writing – review & editing. MK: Conceptualization, Funding acquisition, Resources, Supervision, Validation, Visualization, Writing – original draft, Writing – review & editing. SH: Conceptualization, Methodology, Supervision, Validation, Visualization, Writing – original draft, Writing – review & editing.

## References

[ref1] Abdelazeem K. N. M. Kalo M. Z. Beer-Hammer S. Lang F. (2021). The gut microbiota metabolite urolithin A inhibits NF-κB activation in LPS stimulated BMDMs. Sci. Rep. 11:7117. doi: 10.1038/s41598-021-86514-6, PMID: 33782464 PMC8007722

[ref2] Ahsan A. Zheng Y. R. Wu X. L. Tang W. D. Liu M. R. Ma S. J.. (2019). Urolithin A-activated autophagy but not mitophagy protects against ischemic neuronal injury by inhibiting ER stress in vitro and in vivo. CNS Neurosci. Ther. 25, 976–986. doi: 10.1111/cns.13136, PMID: 30972969 PMC6698978

[ref3] Arganda-Carreras I. Fernández-González R. Muñoz-Barrutia A. Ortiz-De-Solorzano C. (2010). 3D reconstruction of histological sections: application to mammary gland tissue. Microsc. Res. Tech. 73, 1019–1029. doi: 10.1002/jemt.20829, PMID: 20232465

[ref4] Barbosa-Silva M. C. Lima M. N. Battaglini D. Robba C. Pelosi P. Rocco P. R. M.. (2021). Infectious disease-associated encephalopathies. Crit. Care 25:236. doi: 10.1186/s13054-021-03659-6, PMID: 34229735 PMC8259088

[ref5] Bjornevik K. Cortese M. Healy B. C. Kuhle J. Mina M. J. Leng Y.. (2022). Longitudinal analysis reveals high prevalence of Epstein-Barr virus associated with multiple sclerosis. Science 375, 296–301. doi: 10.1126/science.abj8222, PMID: 35025605

[ref6] Bowie A. G. Unterholzner L. (2008). Viral evasion and subversion of pattern-recognition receptor signalling. Nat. Rev. Immunol. 8, 911–922. doi: 10.1038/nri2436, PMID: 18989317 PMC7097711

[ref7] Campos A. (2021). Morph data: automating the data extraction process of morphological features of microglial cells in image. J. Bio Rxiv. doi: 10.1101/2021.08.05.455282, PMID: 11309629

[ref8] Chesnokova V. Pechnick R. N. Wawrowsky K. (2016). Chronic peripheral inflammation, hippocampal neurogenesis, and behavior. Brain Behav. Immun. 58, 1–8. doi: 10.1016/j.bbi.2016.01.017, PMID: 26802985 PMC4956598

[ref9] Culbert A. A. Skaper S. D. Howlett D. R. Evans N. A. Facci L. Soden P. E.. (2006). MAPK-activated protein kinase 2 deficiency in microglia inhibits pro-inflammatory mediator release and resultant neurotoxicity. Relevance to neuroinflammation in a transgenic mouse model of Alzheimer disease. J. Biol. Chem. 281, 23658–23667. doi: 10.1074/jbc.M513646200, PMID: 16774924

[ref10] D'amico D. Andreux P. A. Valdés P. Singh A. Rinsch C. Auwerx J. (2021). Impact of the natural compound urolithin A on health, disease, and aging. Trends Mol. Med. 27, 687–699. doi: 10.1016/j.molmed.2021.04.009, PMID: 34030963

[ref11] Dasilva N. A. Nahar P. P. Ma H. Eid A. Wei Z. Meschwitz S.. (2019). Pomegranate ellagitannin-gut microbial-derived metabolites, urolithins, inhibit neuroinflammation in vitro. Nutr. Neurosci. 22, 185–195. doi: 10.1080/1028415X.2017.1360558, PMID: 28784051

[ref12] Disabato D. J. Quan N. Godbout J. P. (2016). Neuroinflammation: the devil is in the details. J. Neurochem. 139, 136–153. doi: 10.1111/jnc.13607, PMID: 26990767 PMC5025335

[ref13] Edler M. K. Mhatre-Winters I. Richardson J. R. (2021). Microglia in aging and Alzheimer’s disease: a comparative species review. Cells 10:1138. doi: 10.3390/cells10051138, PMID: 34066847 PMC8150617

[ref14] Eshraghi M. Adlimoghaddam A. Mahmoodzadeh A. Sharifzad F. Yasavoli-Sharahi H. Lorzadeh S.. (2021). Alzheimer's disease pathogenesis: role of autophagy and Mitophagy focusing in microglia. Int. J. Mol. Sci. 22, 1–36. doi: 10.3390/ijms22073330, PMID: 33805142 PMC8036323

[ref15] Esselun C. Theyssen E. Eckert G. P. (2021). Effects of urolithin A on mitochondrial parameters in a cellular model of early Alzheimer disease. Int. J. Mol. Sci. 22, 1–19. doi: 10.3390/ijms22158333, PMID: 34361099 PMC8347929

[ref16] Fernández-Arjona M. D. M. Grondona J. M. Granados-Durán P. Fernández-Llebrez P. López-Ávalos M. D. (2017). Microglia morphological categorization in a rat model of Neuroinflammation by hierarchical cluster and principal components analysis. Front. Cell. Neurosci. 11:235. doi: 10.3389/fncel.2017.00235, PMID: 28848398 PMC5550745

[ref17] Ferreira T. A. Blackman A. V. Oyrer J. Jayabal S. Chung A. J. Watt A. J.. (2014). Neuronal morphometry directly from bitmap images. Nat. Methods 11, 982–984. doi: 10.1038/nmeth.3125, PMID: 25264773 PMC5271921

[ref18] Foo J. Bellot G. Pervaiz S. Alonso S. (2022). Mitochondria-mediated oxidative stress during viral infection. Trends Microbiol. 30, 679–692. doi: 10.1016/j.tim.2021.12.011, PMID: 35063304

[ref19] Galic M. A. Riazi K. Pittman Q. J. (2012). Cytokines and brain excitability. Front. Neuroendocrinol. 33, 116–125. doi: 10.1016/j.yfrne.2011.12.002, PMID: 22214786 PMC3547977

[ref20] Gasperotti M. Passamonti S. Tramer F. Masuero D. Guella G. Mattivi F.. (2015). Fate of microbial metabolites of dietary polyphenols in rats: is the brain their target destination? ACS Chem. Neurosci. 6, 1341–1352. doi: 10.1021/acschemneuro.5b00051, PMID: 25891864

[ref21] Gong Z. Huang J. Xu B. Ou Z. Zhang L. Lin X.. (2019). Urolithin A attenuates memory impairment and neuroinflammation in APP/PS1 mice. J. Neuroinflammation 16:62. doi: 10.1186/s12974-019-1450-3, PMID: 30871577 PMC6417212

[ref22] He Y. Taylor N. Yao X. Bhattacharya A. (2021). Mouse primary microglia respond differently to LPS and poly (I:C) in vitro. Sci. Rep. 11:10447. doi: 10.1038/s41598-021-89777-1, PMID: 34001933 PMC8129154

[ref23] Hernangómez M. Mestre L. Correa F. G. Loría F. Mecha M. Iñigo P. M.. (2012). CD200-CD200R1 interaction contributes to neuroprotective effects of anandamide on experimentally induced inflammation. Glia 60, 1437–1450. doi: 10.1002/glia.22366, PMID: 22653796

[ref24] Hosking M. P. Lane T. E. (2010). The role of chemokines during viral infection of the CNS. PLoS Pathog. 6:e1000937. doi: 10.1371/journal.ppat.1000937, PMID: 20686655 PMC2912390

[ref25] Hosseini S. Korte M. (2023). How viral infections cause neuronal dysfunction: a focus on the role of microglia and astrocytes. Biochem. Soc. Trans. 51, 259–274. doi: 10.1042/BST20220771, PMID: 36606670

[ref26] Hosseini S. Michaelsen-Preusse K. Schughart K. Korte M. (2021). Long-term consequence of non-neurotropic H3N2 influenza a virus infection for the progression of Alzheimer's disease symptoms. Front. Cell. Neurosci. 15:643650. doi: 10.3389/fncel.2021.643650, PMID: 33994946 PMC8113629

[ref27] Hosseini S. Wilk E. Michaelsen-Preusse K. Gerhauser I. Baumgärtner W. Geffers R.. (2018). Long-term Neuroinflammation induced by influenza a virus infection and the impact on hippocampal neuron morphology and function. J. Neurosci. 38, 3060–3080. doi: 10.1523/JNEUROSCI.1740-17.2018, PMID: 29487124 PMC6596076

[ref28] Jang H. Boltz D. Mcclaren J. Pani A. K. Smeyne M. Korff A.. (2012). Inflammatory effects of highly pathogenic H5N1 influenza virus infection in the CNS of mice. J. Neurosci. 32, 1545–1559. doi: 10.1523/JNEUROSCI.5123-11.2012, PMID: 22302798 PMC3307392

[ref29] Jang H. Boltz D. Sturm-Ramirez K. Shepherd K. R. Jiao Y. Webster R.. (2009). Highly pathogenic H5N1 influenza virus can enter the central nervous system and induce neuroinflammation and neurodegeneration. Proc. Natl. Acad. Sci. USA 106, 14063–14068. doi: 10.1073/pnas.0900096106, PMID: 19667183 PMC2729020

[ref30] Jorgačevski J. Potokar M. (2023). Immune functions of astrocytes in viral Neuroinfections. Int. J. Mol. Sci. 24, 1–20. doi: 10.3390/ijms24043514, PMID: 36834929 PMC9960577

[ref31] Jurga A. M. Paleczna M. Kuter K. Z. (2020). Overview of general and discriminating markers of differential microglia phenotypes. Front. Cell. Neurosci. 14:198. doi: 10.3389/fncel.2020.00198, PMID: 32848611 PMC7424058

[ref32] Karperien A. L. (2013). Fraclac for ImageJ. Albury-Wodonga: Charles Sturt University. 1–36.

[ref33] Kaul M. Garden G. A. Lipton S. A. (2001). Pathways to neuronal injury and apoptosis in HIV-associated dementia. Nature 410, 988–994. doi: 10.1038/35073667, PMID: 11309629

[ref34] Khalil B. A. Elemam N. M. Maghazachi A. A. (2021). Chemokines and chemokine receptors during COVID-19 infection. Comput. Struct. Biotechnol. J. 19, 976–988. doi: 10.1016/j.csbj.2021.01.034, PMID: 33558827 PMC7859556

[ref35] Klein R. S. Garber C. Funk K. E. Salimi H. Soung A. Kanmogne M.. (2019). Neuroinflammation during RNA viral infections. Annu. Rev. Immunol. 37, 73–95. doi: 10.1146/annurev-immunol-042718-041417, PMID: 31026414 PMC6731125

[ref36] Leyh J. Paeschke S. Mages B. Michalski D. Nowicki M. Bechmann I.. (2021). Classification of microglial morphological phenotypes using machine learning. Front. Cell. Neurosci. 15:701673. doi: 10.3389/fncel.2021.701673, PMID: 34267628 PMC8276040

[ref37] Li L. Mao S. Wang J. Ding X. Zen J. Y. (2019). Viral infection and neurological disorders—potential role of extracellular nucleotides in neuroinflammation. ExRNA 1:26. doi: 10.1186/s41544-019-0031-z

[ref38] Lier J. Streit W. J. Bechmann I. (2021). Beyond activation: characterizing microglial functional phenotypes. Cells 10, 1–13. doi: 10.3390/cells10092236, PMID: 34571885 PMC8464670

[ref39] Lima M. N. Barbosa-Silva M. C. Maron-Gutierrez T. (2022). Microglial priming in infections and its risk to neurodegenerative diseases. Front. Cell. Neurosci. 16:878987. doi: 10.3389/fncel.2022.878987, PMID: 35783096 PMC9240317

[ref40] Liu M. Chen F. Liu T. Chen F. Liu S. Yang J. (2017). The role of oxidative stress in influenza virus infection. Microbes Infect. 19, 580–586. doi: 10.1016/j.micinf.2017.08.008 28918004

[ref41] Lonnemann N. Hosseini S. Marchetti C. Skouras D. B. Stefanoni D. D'alessandro A.. (2020). The NLRP3 inflammasome inhibitor OLT1177 rescues cognitive impairment in a mouse model of Alzheimer's disease. Proc. Natl. Acad. Sci. USA 117, 32145–32154. doi: 10.1073/pnas.2009680117, PMID: 33257576 PMC7749353

[ref42] Michaelsen K. Zagrebelsky M. Berndt-Huch J. Polack M. Buschler A. Sendtner M.. (2010). Neurotrophin receptors TrkB.T1 and p75NTR cooperate in modulating both functional and structural plasticity in mature hippocampal neurons. Eur. J. Neurosci. 32, 1854–1865. doi: 10.1111/j.1460-9568.2010.07460.x, PMID: 20955473

[ref43] Mogensen T. H. Paludan S. R. (2005). Reading the viral signature by toll-like receptors and other pattern recognition receptors. J. Mol. Med. (Berl) 83, 180–192. doi: 10.1007/s00109-004-0620-6, PMID: 15635478

[ref44] Neumann H. Kotter M. R. Franklin R. J. (2009). Debris clearance by microglia: an essential link between degeneration and regeneration. Brain 132, 288–295. doi: 10.1093/brain/awn109, PMID: 18567623 PMC2640215

[ref45] Nimmerjahn A. Kirchhoff F. Helmchen F. (2005). Resting microglial cells are highly dynamic surveillants of brain parenchyma in vivo. Science 308, 1314–1318. doi: 10.1126/science.1110647, PMID: 15831717

[ref46] Paolicelli R. C. Sierra A. Stevens B. Tremblay M. E. Aguzzi A. Ajami B.. (2022). Microglia states and nomenclature: a field at its crossroads. Neuron 110, 3458–3483. doi: 10.1016/j.neuron.2022.10.020, PMID: 36327895 PMC9999291

[ref47] Perry V. H. Holmes C. (2014). Microglial priming in neurodegenerative disease. Nat. Rev. Neurol. 10, 217–224. doi: 10.1038/nrneurol.2014.38 24638131

[ref48] Pliss A. Kuzmin A. N. Prasad P. N. Mahajan S. D. (2022). Mitochondrial dysfunction: a prelude to Neuropathogenesis of SARS-CoV-2. ACS Chem. Neurosci. 13, 308–312. doi: 10.1021/acschemneuro.1c00675, PMID: 35049274 PMC8790819

[ref49] Prinz M. Masuda T. Wheeler M. A. Quintana F. J. (2021). Microglia and central nervous system-associated macrophages-from origin to disease modulation. Annu. Rev. Immunol. 39, 251–277. doi: 10.1146/annurev-immunol-093019-110159, PMID: 33556248 PMC8085109

[ref50] Qiu J. Chen Y. Zhuo J. Zhang L. Liu J. Wang B.. (2022). Urolithin A promotes mitophagy and suppresses NLRP3 inflammasome activation in lipopolysaccharide-induced BV2 microglial cells and MPTP-induced Parkinson's disease model. Neuropharmacology 207:108963. doi: 10.1016/j.neuropharm.2022.108963, PMID: 35065082

[ref51] Reshi M. L. Su Y. C. Hong J. R. (2014). RNA viruses: ROS-mediated cell death. Int J Cell Biol 2014:467452, 1–16. doi: 10.1155/2014/467452 24899897 PMC4034720

[ref52] Ryu D. Mouchiroud L. Andreux P. A. Katsyuba E. Moullan N. Nicolet-Dit-Félix A. A.. (2016). Urolithin A induces mitophagy and prolongs lifespan in C. Elegans and increases muscle function in rodents. Nat. Med. 22, 879–888. doi: 10.1038/nm.4132, PMID: 27400265

[ref53] Sadasivan S. Zanin M. Obrien K. Schultz-Cherry S. Smeyne R. J. (2015). Induction of microglia activation after infection with the non-neurotropic a/CA/04/2009 H1N1 influenza virus. PLoS One 10:e0124047. doi: 10.1371/journal.pone.0124047, PMID: 25861024 PMC4393251

[ref54] Sahu P. S. Ter E. (2018). Interactions between neurotropic pathogens, neuroinflammatory pathways, and autophagic neural cell death. Neuroimmunol. Neuroinflam. 5:2. doi: 10.20517/2347-8659.2017.43

[ref55] Streit W. J. Mrak R. E. Griffin W. S. (2004). Microglia and neuroinflammation: a pathological perspective. J. Neuroinflammation 1:14. doi: 10.1186/1742-2094-1-14, PMID: 15285801 PMC509427

[ref56] Streit W. J. Walter S. A. Pennell N. A. (1999). Reactive microgliosis. Prog. Neurobiol. 57, 563–581. doi: 10.1016/S0301-0082(98)00069-0 10221782

[ref57] Sumien N. Cunningham J. T. Davis D. L. Engelland R. Fadeyibi O. Farmer G. E.. (2021). Neurodegenerative disease: roles for sex, hormones, and oxidative stress. Endocrinology 162, 1–14. doi: 10.1210/endocr/bqab185 PMC8462383 34467976

[ref58] Thangaraj A. Periyasamy P. Liao K. Bendi V. S. Callen S. Pendyala G.. (2018). HIV-1 TAT-mediated microglial activation: role of mitochondrial dysfunction and defective mitophagy. Autophagy 14, 1596–1619. doi: 10.1080/15548627.2018.1476810, PMID: 29966509 PMC6135576

[ref59] Town T. Jeng D. Alexopoulou L. Tan J. Flavell R. A. (2006). Microglia recognize double-stranded RNA via TLR3. J. Immunol. 176, 3804–3812. doi: 10.4049/jimmunol.176.6.3804, PMID: 16517751

[ref60] Velagapudi R. Lepiarz I. El-Bakoush A. Katola F. O. Bhatia H. Fiebich B. L.. (2019). Induction of autophagy and activation of SIRT-1 deacetylation mechanisms mediate neuroprotection by the pomegranate metabolite Urolithin A in BV2 microglia and differentiated 3D human neural progenitor cells. Mol. Nutr. Food Res. 63:e1801237. doi: 10.1002/mnfr.201801237 30811877

[ref61] Vidal-Itriago A. Radford R. A. W. Aramideh J. A. Maurel C. Scherer N. M. Don E. K.. (2022). Microglia morphophysiological diversity and its implications for the CNS. Front. Immunol. 13:997786. doi: 10.3389/fimmu.2022.997786, PMID: 36341385 PMC9627549

[ref62] Woodburn S. C. Bollinger J. L. Wohleb E. S. (2021). The semantics of microglia activation: neuroinflammation, homeostasis, and stress. J. Neuroinflammation 18:258. doi: 10.1186/s12974-021-02309-6, PMID: 34742308 PMC8571840

[ref63] Xu Y. Jin M. Z. Yang Z. Y. Jin W. L. (2021). Microglia in neurodegenerative diseases. Neural Regen. Res. 16, 270–280. doi: 10.4103/1673-5374.290881, PMID: 32859774 PMC7896205

[ref64] Xu J. Yuan C. Wang G. Luo J. Ma H. Xu L.. (2018). Urolithins attenuate LPS-induced Neuroinflammation in BV2Microglia via MAPK, Akt, and NF-κB signaling pathways. J. Agric. Food Chem. 66, 571–580. doi: 10.1021/acs.jafc.7b03285, PMID: 29336147

