# Supplementary material for: The effects of urolithin A on poly I:C-induced microglial activation
Source: Front Cell Neurosci. 2024 Mar 20;18:1343562. doi: 10.3389/fncel.2024.1343562 (PMC10993698; doi:10.3389/fncel.2024.1343562)
Supplement: Supplementary file 1 [file Table_1.DOCX]

Purity of cultures:


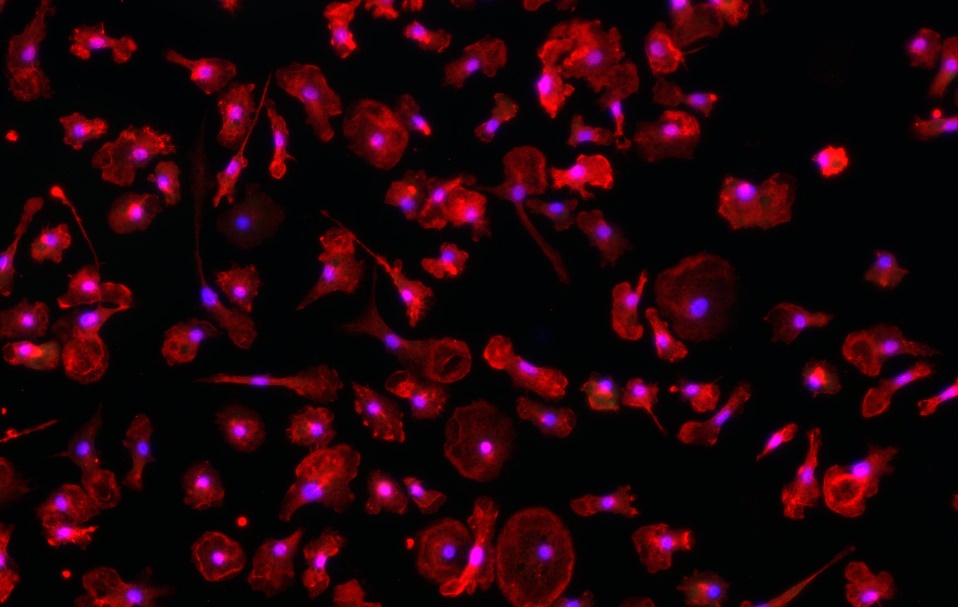


Pure microglia culture: IBA1 (red), DAPI (blue) and GFAP (green), no GFAP staining visible, so the purity is validated.


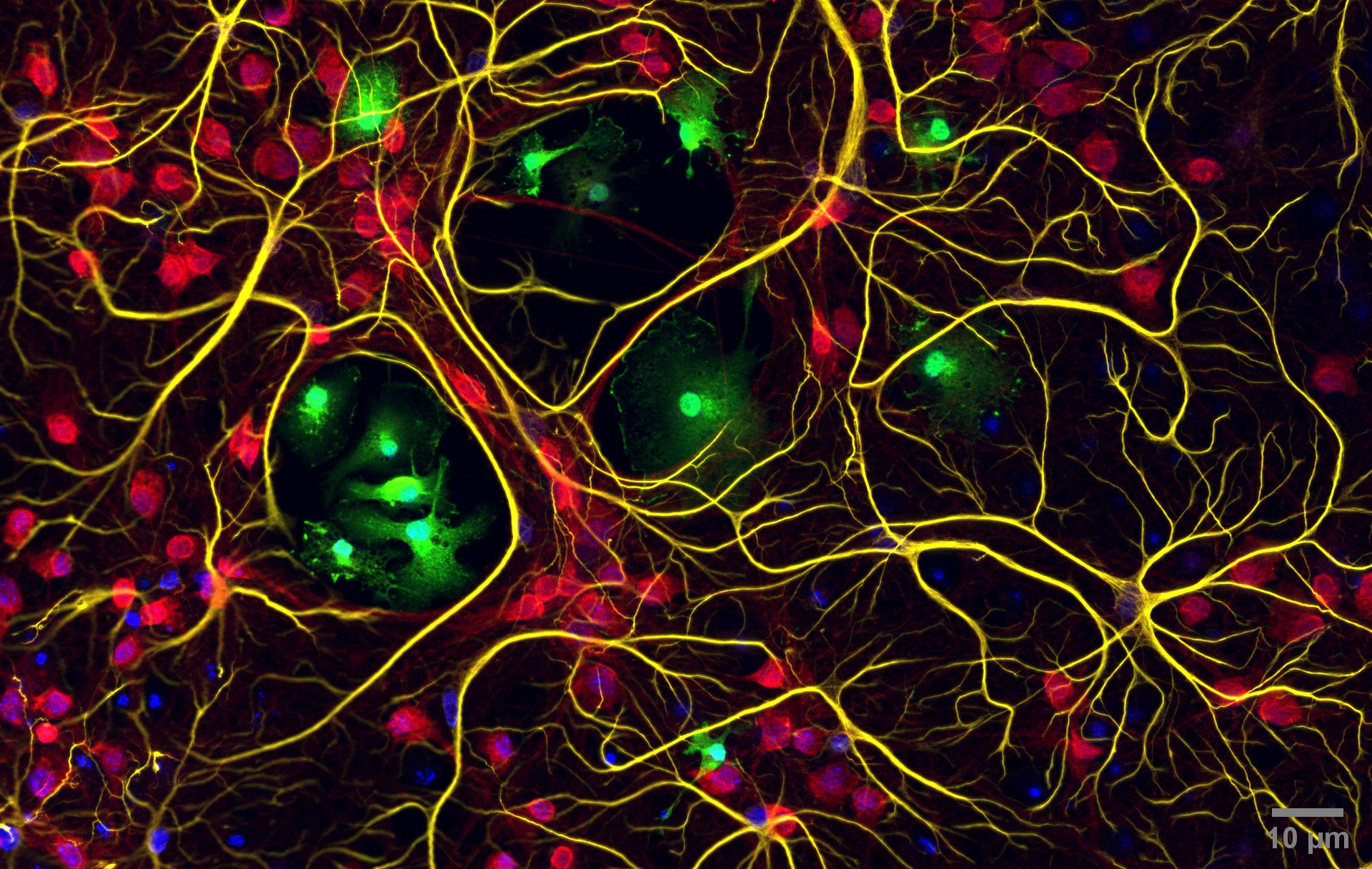


Triple co-culture: IBA1 (green), NeuN (red) and GFAP (yellow), the purity is validated.
